# Beyond Arabidopsis: Differential UV-B Response Mediated by UVR8 in Diverse Species

**DOI:** 10.3389/fpls.2019.00780

**Published:** 2019-06-18

**Authors:** Vanesa Eleonora Tossi, Jose Javier Regalado, Jesica Iannicelli, Leandro Ezequiel Laino, Hernan Pablo Burrieza, Alejandro Salvio Escandón, Sandra Irene Pitta-Álvarez

**Affiliations:** ^1^Laboratorio de Cultivo Experimental de Plantas y Microalgas, Departamento de Biodiversidad y Biología Experimental, Facultad de Ciencias Exactas y Naturales, Universidad de Buenos Aires, Buenos Aires, Argentina; ^2^Instituto de Micología y Botánica, CONICET-Universidad de Buenos Aires, Buenos Aires, Argentina; ^3^Instituto de Genética “Ewald A. Favret,” Instituto Nacional de Tecnología Agropecuaria, Buenos Aires, Argentina; ^4^CONICET-Consejo Nacional de Investigaciones Científicas y Tecnológicas, Buenos Aires, Argentina; ^5^Laboratorio de biología del desarrollo de las plantas, Departamento de Biodiversidad y Biología Experimental, Facultad de Ciencias Exactas y Naturales, Universidad de Buenos Aires, Buenos Aires, Argentina; ^6^Instituto de Biodiversidad y Biología Experimental, CONICET-Universidad de Buenos Aires, Buenos Aires, Argentina

**Keywords:** UV-B, UVR8, RUP1, RUP2, Arabidopsis, terrestrial plants, photomorphogenic response, gene expression

## Abstract

Ultraviolet-B radiation (UV-B, 280–315 nm) is an important environmental signal that regulates growth and development in plants. Two dose-dependent UV-B response pathways were described in plants: a specific one, mediated by UVR8 (the specific UV-B receptor) and an unspecific one, activated by the oxidative damage produced by radiation. The constitutively expressed receptor appears inactive as a dimer, with the two monomers dissociating upon UV-B irradiation. The monomer then interacts with COP1, an ubiquitin ligase, hindering its ability to poly-ubiquitinate transcriptional factor HY5, thus averting its degradation and activating the photomorphogenic response. HY5 induces the synthesis of proteins RUP1 and RUP2, which interact with UVR8, releasing COP1, and inducing the re-dimerization of UVR8. This mechanism has been thoroughly characterized in Arabidopsis, where studies have demonstrated that the UVR8 receptor is key in UV-B response. Although Arabidopsis importance as a model plant many mechanisms described in this specie differ in other plants. In this paper, we review the latest information regarding UV-B response mediated by UVR8 in different species, focusing on the differences reported compared to Arabidopsis. For instance, UVR8 is not only induced by UV-B but also by other agents that are expressed differentially in diverse tissues. Also, in some of the species analyzed, proteins with low homology to RUP1 and RUP2 were detected. We also discuss how UVR8 is involved in other developmental and stress processes unrelated to UV-B. We conclude that the receptor is highly versatile, showing differences among species.

## Introduction

Approximately 7% of all solar radiation that reaches the Earth's surface is ultraviolet light (UV) (Frohnmeyer and Staiger, 2003). UV radiation is divided into three wavelength ranges: UV-A (315–400 nm), UV-B (280–315 nm) and UV-C (100–280 nm) (Björn, 2015). UV-C, the most energetic of the three, is completely absorbed by the ozone (O_3_) layer. UV-A is not attenuated by ozone, but it is the least damaging and acts as a photomorphogenic signal (Björn, 1996). Approximately 95% of UV-B radiation is absorbed by the ozone layer and reaches the Earth's surface with an average intensity of 1 W.m^−2^ (Cejka et al., 2011). Depending on the dosage, UV-B radiation can either act as a signal or cause damage. Due to its high energy, UV-B can break chemical bonds and produce highly reactive molecules.

The natural dynamic balance of ozone levels was abruptly interrupted by anthropogenic emission of ozone-depleting substances (ODS) (from the 1960's to the end of the 1990's), with a decrease in ozone levels and an increase in UV-B radiation reaching the Earth's surface (Steinbrecht et al., 2018). Thirty years after the Montreal Treaty, which banned ODS emission worldwide, the ozone layer shows signs of recovery (Ball et al., 2018; Steinbrecht et al., 2018). Nonetheless, this recovery has not been reported in the areas where practically all inhabitants and cultivars are located Chipperfield et al. (2017), Weber et al. (2018). Some papers have even reported a decrease in stratospheric ozone in these latitudes since 1998 (Ball et al., 2018).

The most common effects induced by UV-B radiation in plants are biomass decrease (Tevini and Teramura, 1989; Vandenbussche et al., 2018), alterations in cuticle and epidermis (Tevini and Steinmüller, 1987), abnormal growth (Teramura and Sullivan, 1994; Searles et al., 2001), and damages to photosystems I and II (Pang and Hays, 1991; Liu et al., 2013). These effects can affect both cultivar quality and yield (Bais et al., 2018). It has been estimated that cultivar growth decreases approximately 1% for each 3% increase in UV-B radiation (Ballaré et al., 2011), but this could be more dramatic in UV-B sensitive cultivars (Hakala et al., 2002; Gao et al., 2003; Hidema and Kumagai, 2006; Zhua and Yang, 2015). UV-B radiation could also have effects on food quality, by affecting levels and composition of vitamins, fatty acids, polyphenols, and flavonoids or anthocyanins, thus modifying nutritional and organoleptic characteristics (Choudhary and Agrawal, 2016; Reddy et al., 2016; Tripathi and Agrawal, 2016; Nguyen et al., 2017; Wang et al., 2017; Wu et al., 2017).

Knowing how plants react to UV-B radiation is therefore crucial to establish damage reducing strategies for cultivars. In the past 20 years, there have been great advances in this area, but most of the work has been done using *Arabidopsis thaliana* as a model plant. One of the outstanding advances has been the characterization of the first specific UV-B photoreceptor: UV RESISTANCE LOCUS 8 (UVR8) (Rizzini et al., 2011; Christie et al., 2012; Wu et al., 2012).

The aim of this article is to review the current documented knowledge concerning plants' responses to UV-B radiation, venturing beyond the information available for *Arabidopsis thaliana* and focusing on other species. To achieve this, we spotlighted the responses mediated by the UVR8 receptor and the UV-B response repressors, RUP proteins (REPRESSOR OF UV-B PHOTOMORPHOGENESIS). Furthermore, we analyzed the participation of UVR8 in diverse stresses, fruit development, and UV-B-independent responses.

## UVR8-mediated UV-B Signaling in Arabidopsis

**Figure 1:**
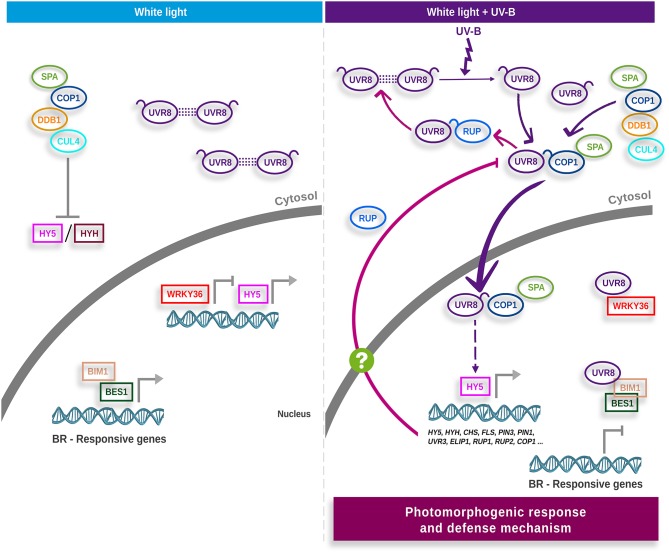
UVR8-mediated signal transduction model in Arabidopsis. In white light **(left panel)**, the UV-B photoreceptor UVR8 homodimer and E3 ubiquitin ligase complex are located in the cytosol. The E3 ubiquitin ligase promotes degradation of the HY5 and HYH transcription factors. HY5, acting redundantly with HYH, mediates transcriptional responses. Transcription factors HY5, WRKY36, BIM1 and the functional BES1 are localized in the nucleus. HY5 binds to its own promoter to activate HY5 transcription, and WRKY36 also binds to the HY5 promoter to inhibit its transcription. BIM1 and BES1 act together to induce the expression of brassinosteroid (BR)-responsive genes. When plants are exposed to UV-B **(right panel)**, the UVR8 homodimer is dissociated into active monomers. Monomeric UVR8 binds to COP1–SPA and elicits the COP1–SPA dissociation from the CUL4–DDB1 E3 ubiquitin ligase complex precluding HY5/HYH degradation. UVR8–COP1–SPA travels to the nucleus and facilitates HY5 protein stabilization and enhances the binding of HY5 to its own promoter. UVR8 monomer interacts with WRKY36 to inhibit WRKY36 binding to the HY5 promoter hence removing the inhibition of HY5 expression. In addition, the UVR8 monomer interacts with BIM1 and the functional dephosphorylated BES1 to inhibit their binding to the BR-induced genes involved in cell elongation, thus repressing the expression of BR-induced elongation genes and further repressing the BR-promoted plant growth. HY5 induces the transcription of key genes in the photomorphogenic response and defense mechanism. RUP1 y RUP2 are two of the genes induced by HY5. RUP proteins (RUP1 and RUP2) negatively regulate UVR8 by binding to the C27 region, displacing COP1, and promoting re-dimerization of the photoreceptor (pink arrows). RUP could act both in the nucleus and in the cytosol. Currently, the mechanism that transports RUP between the cytosol and the nucleus is unknown. The figure is based on the models proposed by Jenkins (2017) and Liang et al. (2019). UV-B, ultraviolet-B radiation; UVR8, UV RESISTANCE LOCUS 8; COP1, CONSTITUTIVELY PHOTOMORPHOGENIC 1; HY5, ELONGATED HYPOCOTYL 5; HYH, HY5 HOMOLOG; SPA1, SUPPRESSOR OF PHYA; DDB1, DNA DAMAGE-BINDING PROTEIN 1; CUL4, CULLIN 4; WRKY36, WRKY DNA-BINDING PROTEIN 36; BES1, BRI1-EMS-SUPPRESSOR1; BIM1, BES1-INTERACTING MYC-LIKE 1; BRs, Brassinosteroids; RUP1 and RUP2, REPRESSOR OF UV-B PHOTOMORPHOGENESIS 1 and 2; FLS, FLAVONOL SYNTHASE; UVR3, UV REPAIR DEFECTIVE 3; ELIP1, EARLY LIGHT-INDUCIBLE PROTEIN 1; CHS, CHALCONE SYNTHASE.

Protein UV RESPONSE LOCUS 8 (UVR8) has been characterized as the UV-B radiation receptor (Kliebenstein et al., 2002; Brown et al., 2005; Rizzini et al., 2011; Christie et al., 2012; Jenkins, 2014). As shown in Figure 1, in presence of UV-B, UVR8 changes its quaternary structure from homodimer to active monomer, translocating from the cytoplasm to the nucleus, where it is functional (Kaiserli and Jenkins, 2007; Rizzini et al., 2011; Christie et al., 2012; Qian et al., 2016; Yin et al., 2016). Constitutively photomorphogenic 1 (COP1) is part of the E3 ubiquitin ligase complex, where it interacts with SUPPRESSOR OF PHYA (SPA1), poly-ubiquitinating the transcriptional factors ELONGATED HYPOCOTYL 5 (HY5) and HY5 HOMOLOG (HYH), which are subsequently degraded via proteasome (Lau and Deng, 2012; Huang et al., 2014). In presence of UV-B radiation, the UVR8 monomer interacts with COP1 and disengages COP1-SPA from the E3 ubiquitin ligase complex, avoiding ubiquitination and subsequent degradation of HY5 and HYH (Favory et al., 2009; Cloix et al., 2012). HY5 levels increase, inducing its own expression and regulating the expression of key genes in UV-B response (Brown et al., 2005; Binkert et al., 2014).

One of the genes induced by HY5 encodes for RUP (REPRESSOR OF UV-B PHOTOMORPHOGENESIS) proteins, which participate in the negative feedback regulation by binding to UVR8 and promoting its re-dimerization (Gruber et al., 2010; Heijde and Ulm, 2013). When plants are grown in diurnal periods, UVR8 reaches a dimer/monomer equilibrium in which RUP proteins are crucial for correct dimerization (Findlay and Jenkins, 2016). Other proteins also participate in the negative feedback regulation of UV-B: SALT TOLERANCE/BBX24 (STO/BBX24), RADICAL-INDUCED CELL DEATH1 (RCD1) and DWD HYPERSENSITIVE TO UV-B 1 (DHU1) (Jiang et al., 2009, 2012a,b; Kim et al., 2017). BBX24 binds to RCD1 and interacts with HY5 and COP1, stabilizing COP1, reducing HY5 accumulation and inhibiting its activity as a transcriptional factor (Jiang et al., 2012a). DHU1 sequesters COP1, found in the complex UVR8-COP1, diminishing the UV-B response (Kim et al., 2017).

On the other hand, it has been demonstrated that UVR8 monomers can interact with the transcriptional factors WRKY DNA-BINDING PROTEIN 36 (WRKY36), BRI1-EMS-SUPPRESSOR1 (BES1) and BES1-INTERACTINGMYC-LIKE 1 (BIM1) in the nucleus, as part of a signaling cascade in response to UV-B (Favory et al., 2009; Liang et al., 2018; Yang et al., 2018a). Free WRKY36 binds to the HY5 promoter and blocks its transcription. When UVR8 interacts with WRKY36, this factor is removed and HY5 can induce its expression (Yang et al., 2018a). BES1 and BIM1 are part of the Brassinosteroids' (BRs) signaling cascade (Yin et al., 2005). BES 1 interacts with BIM1 and binds to DNA, inducing BRs response genes (Yin et al., 2005; Belkhadir and Jaillais, 2015; Liang et al., 2018). The monomer UVR8 binds preferentially to dephosphorylated BES1, which is the active form, and by precluding its interaction with DNA, promotes one of the classic responses to UV-B: the inhibition of hypocotyl growth (Vert and Chory, 2006; Jiang et al., 2015; Liang et al., 2018).

## UVR8: from Green Algae to Higher Plants

UVR8 is functional as a receptor because of amino acid sequences that are key for UV-B perception, homodimerization, and COP1 interaction. UV-B perception is achieved through tryptophan residues (O'Hara and Jenkins, 2012; Ulm and Jenkins, 2015). UVR8 monomers have three Gly-Trp-Arg-His-Thr motifs (GWRHT) that form a tryptophan triade (W233, W285, and W337), where W285 is the main UV-B sensor. Once the tryptophans absorb UV-B radiation, the dimer dissociates into its monomers (Christie et al., 2012; Wu et al., 2012). The interaction of UVR8 with COP1 may occur either in a UV-B dependent fashion, through a β-propeller domain, or constitutively, via core VP (Val-Pro) in the UVR8C27 domain (Cloix et al., 2012; Yin et al., 2015). By analyzing the amino acid sequences and focusing on the key motifs for UVR8 function, UVR8 sequences have been identified in green algae, bryophytes, lycophytes, and angiosperms (Rizzini et al., 2011; Wu et al., 2012; Fernández et al., 2016). These motifs are highly conserved from green algae to higher plants. At the moment, no UVR8 homologs in gymnosperms have been found or characterized, making it an issue worth exploring. Characterization studies for the UVR8 receptor have been performed in Arabidopsis (Rizzini et al., 2011; Cloix et al., 2012; Wu et al., 2012), and, in recent years, studies of UVR8 in other species have been undertaken. In green algae, such as *Chlamydomonas reinhardtii* (Cr), CrUVR8 dimers dissociate upon UV-B irradiation and re-form when transferred to white light. Interaction between CrUVR8 and CrCOP1 in *Chlamydomonas* has been confirmed in yeast two-hybrid assays. In addition, CrUVR8 complements the Arabidopsis *uvr8* mutant, indicating that the molecular mechanism of action characterized in Arabidopsis is well-conserved (Tilbrook et al., 2016).

Soriano et al. (2018) studied the structure and function of UVR8 in two bryophytes, the moss *Physcomitrella patens* and the liverwort *Marchantia polymorpha*. Unlike Arabidopsis, which only has one *UVR8* gene, *P. patens* expresses two *UVR8* genes that encode functional proteins, whereas the single *M. polymorpha UVR8* gene expresses two transcripts by alternative splicing, and the resulting proteins are functional. In *Physcomitrella*, the UVR8 dimer also dissociates in the presence of UV-B (as in Arabidopsis), but in its absence, both dimers and monomers were detected. However, in *M. polymorpha*, the UVR8 monomer is present independently of UV-B treatment and the dimer UVR8 is barely detected.

UVR8 is translocated from the cytoplasm into the nucleus, where it accumulates rapidly when Arabidopsis plants are exposed to UV-B (Kaiserli and Jenkins, 2007; Yin et al., 2016). Contrary to Arabidopsis, both *M. polymorpha* GFP-UVR8 fusion proteins encoded in *uvr8* are found in the nucleus, independently of UV-B treatment. Nevertheless, both *Marchantia* and *Physcomitrella* UVR8 complement Arabidopsis *uvr8* mutants, thus reinstating HY5 transcript levels, the accumulation of the chalcone-synthase enzyme (CHS), and the suppression of hypocotyl growth in response to UV-B.

In Angiosperms such as *Chrysanthemum morifolium* (Cm), *Populus euphratica* (Pe), and *Malus domestica* (Md), heterologous expression of CmUVR8, PeUVR8, and MdUVR8 rescued the deficient phenotype of *uvr8* mutants in Arabidopsis in response to UV-B. In addition, the CmUVR8-CmCOP1, and MdUVR8-MdCOP1 interactions were confirmed using a yeast two-hybrid assay. PeUVR8-AtCOP1 interaction was verified using bimolecular fluorescence complementation (BiFC) assay (Mao et al., 2015; Zhao et al., 2016; Yang et al., 2018b).

In agreement with previous studies in Arabidopsis, overexpression of UVR8 in *Solanum lycopersicum* (Sl) increased tolerance to UV-B, and gene silencing in tomato lines (SlUVR8Ri) promoted UV-B hypersensibility (Li H. et al., 2018). UV-B response gene induction, such as *HY5* and *CHS*, as well as anthocyanin accumulation were repressed in SlUVR8Ri, indicating that SlUVR8 plays an essential role in UV-B response. GFP-SlUVR8 proteins expressed in *Nicotiana* showed cytoplasm to nucleus translocation as reported for Arabidopsis (Li H. et al., 2018).

In all the species mentioned, the UVR8 receptor was functional and behaved similarly to the receptor described for Arabidopsis. This confirms the predicted presence of UVR8 in green algae, bryophytes, and angiosperms by bioinformatics analyses. The existence of UVR8 in ancestral organisms, such as green algae, could be related to the elevated UV-B dose in the primitive atmosphere and the ensuing self-protection required by the first photosynthetic plants (Jenkins, 2017).

## UVR8 Expression in Different Plant Species

The expression of the *UVR8* gene in Arabidopsis (At*UVR8*) is ubiquitous and constitutive in plant tissues, and this includes roots, which do not receive direct light (Rizzini et al., 2011; Jenkins, 2017). Arabidopsis has only one copy of the *UVR8* gene, while several species contain at least two UVR8 genes (Brown et al., 2005; Fernández et al., 2016). Although, at the moment, there is no evidence of functional differences between UVR8 proteins within analyzed species, the possession of multiple genes provides the potential for differential expression regulation.

As can be observed in Table 1, in species such as *P. euphratica, S. lycopersicum, Betula platyphylla, C. morifolium, or Malus domestica*, the *UVR8* gene is expressed in all the tissues, as in Arabidopsis, but the transcript levels differ significantly between tissues (Mao et al., 2015; Li H. et al., 2018; Yang et al., 2018b). In *Populus*, expression of *PeUVR8* is higher in shoots and leaves, declines in stems, and is weak in roots (Mao et al., 2015). In *Solanum* SlUVR8 expression levels are higher in flowers than in the other tissues analyzed (Li X. et al., 2018). The expression of *BpUVR8* in Betula is highest in leaves and significantly reduced in xylem, phloem, and inflorescences (Li H. et al., 2018). In *Chrysanthemum*, expression is higher in leaves, followed by flowers and weakest in roots. *MdUVR8* gene expression in apple trees is higher in stems, lower in fruits and roots, and very low in leaves and flowers (Zhao et al., 2016). Besides changes in expression levels among apple tissues, the abundance of the transcript *MdUVR8* also varies throughout the development of the fruit (Henry-Kirk et al., 2018). In *Vitis vinifera*, expression of *VvUVR8* in the grape berry varies, and it is four to five times higher in pre-verasion than in post-verasion (Liu et al., 2015).

**Table 1:** *UVR8* gene expression in different organs and developmental stages in diverse species.

**Species**	**Gene**	**Plant Material**	**Type of analysis**	**Expression levels in organs/developmental stages** **(X: times/fold with respect to the tissue with lowest expression)**	**References**
*Arabidopsis thaliana*	*AtUVR8*	Plants	Analysis in different organs	Present in all organs. No significant expression changes among different organs	Kaiserli and Jenkins, 2007; Rizzini et al., 2011
*Betula platyphylla*	*BpUVR8*	Trees grown in natural conditions	Analysis in different organs	Inflorescence (1x); Xylem (1.5x); Phloem (0.75x);Leaves (6.5x)	Li X. et al., 2018
*Chrysanthemum morifolium*	*CmUVR8*	8-month-old plants in natural conditions	Analysis in different organs	Roots (1x); Stems (4.5x); Leaves (6.4x);Flowers (4.5x)	Yang et al., 2018b
*Malus domestica*	*MdUVR8*	Trees grown in natural conditions	Analysis in different organs	Root (1x); Stem (15x); Leaf (0.1x); Flower (0.2x);Fruit (5x)	Zhao et al., 2016
*Populus euphratica*	*PeUVR8*	Trees grown in natural conditions	Analysis in different organs	Roots (1x);Stems (10x);Leaves (22.5x);Shoots (8.5x);Buds (20x)	Mao et al., 2015
*Solanum lycopersicum*	*SlUVR8*	Plants grown in outdoorfield for 4 months	Analysis in different organs	Root (1x); Steam (2.8x); Leaf (7.8x);Flower (9x)	Li X. et al., 2018
*Vitis vinifera*	*VvUVR1*	Grapevine organs of field-grown plants	Analysis in different organs	Seed post veraison (1x); Root (3.7x); Tendril (7.2x); Leaf (5x); Inflorescence (4.5x); Flower (6.3x)	Loyola et al., 2016
*Cucumis sativus*	*CsUVR8*	Hypocotyl of two near isogenic lines	Hypocotyl elongation during the first 15 days of growth	Significant variations in expression levels during the first 15 days	Bo et al., 2016
*Malus x domestica*	*MdUVR8*	Apple peel from 35 days to 146 days after full bloom	Apple ripening	Expression levels change during apple ripening. Expression reaches a maximum 104 days after full bloom	Henry-Kirk et al., 2018
*Solanum lycopersicum*	*SlUVR8*	Fruit pericarp of plants grown in outdoorfields	Fruit development and ripening	Significant variations in fruit: 10 days post-anthesis (3.6x) 20 days post–anthesis (2.4x) breaker fruit (5x); red fruit (3.1 x)	Li H. et al., 2018
*Solanum melongena*	*SmUVR8*	Peels of eggplants grown in horticultural farm	Fruit ripening	Expression levels change during fruit ripening. Expression first increases and later is repressed.	Li H. et al., 2018
*Vitis vinifera*	*VvUVR1*	Berry skin of field-grown plants	Veraison (−4 weeks to +8 weeks)	Expression levels decrease significantly during veraison	Loyola et al., 2016
*Vitis vinifera*	*VvUVR1*	Flowers of field-grown plants	Flower development (−10 weeks before anthesis to anthesis)	Expression levels change significantly during flower maturation, with the lowest values 8 weeks before anthesis	Loyola et al., 2016

The receptor's constitutive presence allows a rapid response to mitigate the damage caused by UV-B (Rizzini et al., 2011). Arabidopsis UVR8 over-expressing mutants are more tolerant and adapt better to UV-B (Favory et al., 2009; Morales et al., 2013). If we assume that higher *UVR8* expression correlates with an increase in protein levels, differential expression in the diverse tissues could be associated with different degrees of UV-B tolerance throughout the plant.

*AtUVR8* expression levels do not vary significantly in response to UV-B (Kliebenstein et al., 2002; Tohge et al., 2011; Fasano et al., 2014). Similarly, *VvUVR8* expression does not change with UV-B treatment in berry skin (Carbonell-Bejerano et al., 2014). Whereas in *M. polymorpha* and *P. patens, UVR8* expression remains constant in presence of UV-B (Soriano et al., 2018), there are some species where *UVR8* expression varies significantly in response to UV-B (see Table 2). In birch tree, after 6 h exposure to UV-B, *BpUVR8* expression increases 3.5 times with respect to the control, with a maximum peak at 9 h (4.65 times more than in the control) (Li J. et al., 2018). In Antarctic moss *Pohlia mutans*, expression levels of genes involved in the UV-B signaling pathway, *PnUVR8, PnHY5* and *PnCOP1*, increase significantly after 3 h treatments with UV-B (Li et al., 2019). In another Antarctic species, such as *Colobanthus quitensis*, the increase in the transcription of *UVR8* and *COP1* is dose-dependent, reaching its peak 6 h after UV-B exposure (Contreras et al., 2019).

**Table 2:** UVR8 gene expression in different plants treated with UV-B.

**Specie**	**Gene**	**UV-B treatment**	**Changes in gene expression** **(X: times/fold with respect to the control)**	**Tissue**	**References**
*Arabidopsis thaliana*	*AtUVR8*	Plants were grown on compost for 3 weeks under 20 μmol m^−2^ s^−1^ constant white light at 21°C and then treated with 3 μmol m^−2^ s^−1^ UV-B for 4 h	No significant changes	Leaf	Kaiserli and Jenkins, 2007
*Arabidopsis thaliana*	*AtUVR8*	Plants were grown *in vitro* for 14 days under white light (100 μmol s^−1^m^−2^) supplemented with UV-B (5 μmol m^−2^s^−1^) for 2 h	No significant changes	Complete plant	Fasano et al., 2014
*Arabidopsis thaliana*	*AtUVR8*	Plants were grown for 10 days before being irradiated with 0.2 kJ UV-BBE m^−2^h^−1^ (or 1.4 kJ m^−2^ h^−1^unweighted UV-B) for 3 d	No significant changes	Leaf	Kliebenstein et al., 2002
*Betula platyphylla*	*BpUVR8*	Seedling were exposed to 1.5 μmol m^−2^ s^−1^ UV-B during 3, 6, 9, 12 and 24 h.	No significant changes in the 3 h treatment. In the remaining treatments, expression increases: 6 h (5.9x) 9 h (7.9x) 12 h (6.5x) and 24 h (2.7x)	3-week-old seedling	Li X. et al., 2018
*Colobanthus quitensis*	*CqUVR8*	Different doses during 24 h: low 1.7 kJ m^−2^d^−1^, medium 15.8 kJ m^−2^d^−1^, andhigh 21.4 kJ m^−2^d^−1^	Reach their highest level 3 h after medium treatment (13x) and 6 h after high treatment (16x)	Complete plant	Contreras et al., 2019
*Cucumis sativus*	*CsUVR8*	~0.15 Jm^−2^ min^−1^ UV irradiation treatment started on the third day after germination with 5 h exposure per day.	No significant changes	Hypocotyl	Bo et al., 2016
*Malus domestica*	*MdUVR8*	Continuous white light (20 μmol m^−2^ s^−1^) supplemented with UV-B (305 nm) (1.5 μmol m^−2^ s^−1^).	Increased progressively and reached a peak at 6 h, but decreased during the next period of time and reached a minimum at 24 h	Fruit skin	Zhao et al., 2016
*Marchantia polymorpha*	*MpUVR8*	Plants were grown under continuous 80 μmol m^−2^ s^−1^ white light. Plants were placed in darkness for 16 h and then exposed to either 20 μmol m^−2^ s^−1^ white light or 3 μmol m^−2^ s^−1^ narrowband UV-B for 3 h	No significant changes	Complete plant	Soriano et al., 2018
*Marchantia polymorpha*	*MpUVR8*	Plants were exposed to different doses of UV-B daily for 12 h: Control: 51 μW cm^−2^ UV-B. Low-fluence UVB treatments: 124 μW cm^−2^ UV-B.	No significant change	Complete plant	Clayton et al., 2018
*Physcomitrella patens*	*PpUVR8.1 PpUVR8.2*	Plants were in dark 16 h and then exposed to 20 μmol m^−2^ s^−1^ white light or 3 μmol m^−2^ s^−1^UV-B (312 nm) 30 min	No significant changes	Complete plant	Soriano et al., 2018
*Pohlia nutans*	*PnUVR8*	UV-B radiation was 0.20 mW cm^−2^ for 3 h or 6 h	*PnUVR8* was significantly after 3h treatments with UV-B	Green gametophyte	Li et al., 2019
*Prunus persica*	*PpUVR8*	Fruits were exposed 10 min to 1.39 kJm^−2^and 60 min to 8.33 kJm^−2^ UV-B	*PpUVR8* expression was not significantly different to the control in both UV-B-treated fruit after exposition, although a slightly higher transcript abundance was detectable 6 h after UV-B exposures	Peach skin	Santin et al., 2019
*Raphanus sativus*	*RsUVR8*	UV-B dose was set at 10 W·m^−2^	2x	Hypocotyl	Wu et al., 2016
*Solanum lycopersicum*	*SlUVR8*	Plants were exposed for 5 min per day to 2.94 kJ/m2 UV-B (312 nm) during 30 days	6x.	Leaf	Mariz-Ponte et al., 2018
*Vitis vinifera*	*VvUVR1*	Plants were exposed for 6 h to 15 μW.cm^−2^ UV-B	No significant changes	Leaf	Loyola et al., 2016
*Vitis vinifera*	*VvUVR8*	Vines were divided into experimental conditions: no filter (Ambient); UV radiation-transmitting filter (FUV+); UV radiation-blocking filter (FUV-).	No significant changes	Fruit skin	Carbonell-Bejerano et al., 2014
*Zea mays*	*ZmUVR8*	5-week old plants were exposed to UV-B (2 W m^−2^) for different periods of time. The first 3 leaves at the top received UV-B radiation while the rest were shielded.	Expression was rapidly increase by UV-B after 10 min in irradiated leaves and shielded leaves. After 4 h of UV-B, expression was down-regulated both in irradiated and shielded leaves	Leaf	Casati et al., 2011a,b

*UVR8* expression is repressed in maize leaves irradiated with UV-B for 1 h (Casati et al., 2011a). However, in the leaves shielded from UV-B, *UVR8* expression is induced as part of a systemic response. The induction observed in the shielded leaves is overturned when the plants are exposed to UV-B more than 4 h (Casati et al., 2011a). In eggplant, as in maize, expression of the UVR8 transcript is induced with 0.5 h UV-B exposure and repressed after 4 and 5 h (Li J. et al., 2018).

In radish sprout hypocotyls, the UVR8 expression is induced significantly by UV-B (2 times with respect to the control) (Wu et al., 2016). Expression levels of *PpUVR8* did not vary substantially in peach skin during treatment with UV-B, but increased slightly after exposure, reaching the highest peak at 6 h (Santin et al., 2019). In conclusion, there are some species in which *UVR8* expression in response to UV-B is constant, as in Arabidopsis, but in most cases the expression levels vary.

## Presence of RUP 1 and RUP2 in Different Species

RUP1, RUP2, and COP1 are members of the WD40-repeat protein family. RUP1 and RUP2 contain seven WD40-repeats with apparently no additional domains (Van Nocker and Ludwig, 2003; Li et al., 2013). In the presence of UV-B, levels of RUP proteins increase and their relative abundance (compared to COP1) interrupts the interaction UVR8-COP1, contributing to the reversal of UVR8 from monomer to homodimer. As a result, RUP proteins switch off the signaling cascade, thus averting problems in plant growth and development (Gruber et al., 2010; Heijde et al., 2013). In plants growing in diurnal photoperiods under natural light, a UVR8 dimer/monomer photo-equilibrium is established. In that state, RUP1 and RUP2 play a crucial role in the reversion of monomer to dimer, allowing for the plant's optimum growth and development in that specific environment (Findlay and Jenkins, 2016).

**Figure 2:**
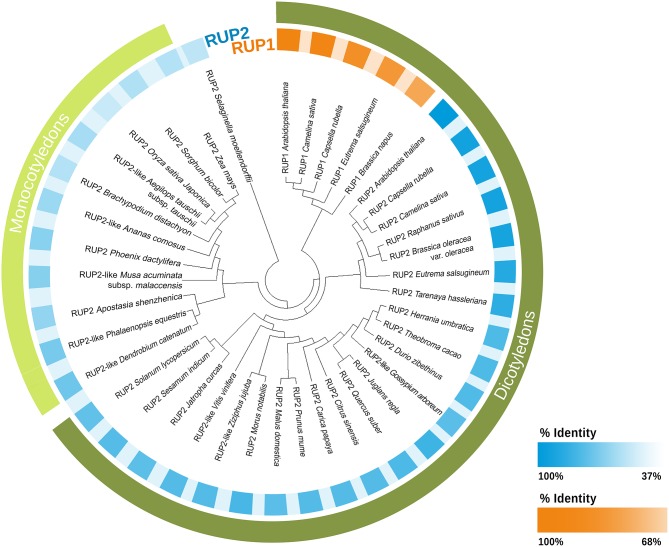
Phylogram of RUP1 and RUP2 proteins. RUPs proteins sequences of plant species and the percentage identity were obtained from the NCBI database (http://www.ncbi.nlm.nih.gov/) using the BLASTP method. The query sequences used were AtRUP1(OAO92149.1) and AtRUP2 (OAO92900.1). The tree was built using Maximum Likelihood method and JTT matrix-based model (Jones et al., 1992). Initial tree obtained by Neighbor-Join and BioNJ algorithms. Distances estimated using JTT. Evolutionary analyses were conducted in MEGA X (Kumar et al., 2018). The highest log likelihood: −15691.88. To make this tree we used some representative sequence of each species. For more information about sequences see Supplementary Table 1.

In addition to RUP proteins, STO/BBX24, RCD1, and DHU1 have also been characterized as negative regulators in the Arabidopsis UV-B signaling cascade (Jiang et al., 2009, 2012a,b; Kim et al., 2017). Most studies concerning the characterization of RUP proteins in response to UV-B have been performed in Arabidopsis (Gruber et al., 2010; Heijde et al., 2013; Vanhaelewyn et al., 2016). AtRUP1 (385aa) and AtRUP2 (368aa) proteins have a 64% identity (Favory et al., 2009; Supplementary Table 1) and both fulfill the same functions in the UV-B response (Vanhaelewyn et al., 2016). In order to search for RUP proteins in terrestrial plants, a blastp (blast protein, ncbi) was carried out using AtRUP1 (OAO92149.1) and AtRUP2 (OAO92900.1) as query sequences. As can be observed in Figure 2 and Supplementary Table 1, there are few RUP1 sequences with a high percent identity (91% to 64%). The sequences identified as RUP1 derive from species of the *Brassicaceae* family, as Arabidopsis. The one exception is a *Tarenaya hassleriana* sequence, which belongs to a *Brassicaceae* sister family. The high percent identity values are to be expected since the sequences derive from the same family.

Recently, in experiments with mutants obtained through CRISPR/Cas9, it has been demonstrated that UV-B response in *M. polymorpha* includes many common components with Arabidopsis, among them RUP1 (Mapoly0094-s0072) (Clayton et al., 2018). In contrast to the RUP1 sequences detected, there is a higher number of RUP2 sequences detected (Figure 2 and Supplementary Table 1). The phylogram shows that RUP1 and RUP2 from monocotyledons and dicotyledons belong to different clusters (Figure 2).

The percent identity in the sequences identified as RUP2 in dicotyledons (93%-53%) is higher than in monocotyledons (lower than 55%). Due to the low percent identity of RUP2 proteins in monocotyledons and the fact that RUP1 sequences have not been detected in this group, it could be hypothesized that RUP proteins would not be part of the switch-off mechanism in monocotyledons.

One hypothesis is that mechanisms such as the ones mediated by STO/BBX24 and DHU1 suffice to switch off the UV-B response. Another possibility is the existence of proteins fulfilling the same functions as RUPs, but with a low percent identity. Yet another proposition is that the negative regulation mechanism could be based on direct repression of UVR8 expression or UVR8 monomer degradation, whenever necessary. Research has demonstrated that, in several species, *UVR8* gene expression is regulated in the presence of UV-B (Table 2). In maize, for example, Casati et al. (2011a) reported that, after extended exposure to UV-B, UVR8 expression was repressed both in exposed and shielded leaves, possibly as part of negative feedback.

There are proteins that have a low percent identity but still exhibit conserved domains that allow them to fulfill the same functions. RUP proteins have seven WD40-repeats with apparently no additional domains (Gruber et al., 2010). It was observed that the C27 region of UVR8 interacts with the WD40 region of COP1 and RUP1/2 (Gruber et al., 2010; Cloix et al., 2012). It would be interesting to examine the degree of conservation of the WD40 domain among RUP2 proteins in monocotyledons and dicotyledons, and if it can interact with UVR8 as it does in Arabidopsis.

## UVR8 in Fruit Developmental Stages

UV-B is an environmental signal that is perceived by plants, modulating growth, development, and metabolism in different organs (Tilbrook et al., 2013). By studying Arabidopsis mutants, the participation of UVR8 in many of the responses triggered by UV-B in leaf (Wargent et al., 2009), hypocotyl (Liang et al., 2019), root (Wan et al., 2018), and flower (Dotto et al., 2018) has been determined, but further research in fruit is required. UV-B promotes flavonoids and anthocyanins accumulation in Arabidopsis and other species (Lois, 1994; Ubi et al., 2006). In Arabidopsis leaves, synthesis of flavonoids and anthocyanins in response to solar UV-B radiation is regulated by the UVR8 receptor (Morales et al., 2013). The production of other pigments, such as carotenoids, is also induced by UV-B (Becatti et al., 2009). Furthermore, UV-B radiation increases fruit color in several fruit trees like grapes, apples, peaches, and blueberries, among others (Zhao et al., 2016; Escobar et al., 2017; Henry-Kirk et al., 2018). Fruit nutritional quality frequently depends on the content of those pigments, and their composition varies throughout the fruit's development and ripening (Wang et al., 2008).

In sunlight conditions, anthocyanins accumulate in the pericarp of *Litchi chinensis*. In *Litchi*, eight putative *UVR8* encoding genes were identified, and their expression increases differentially when the fruit is exposed to UV radiation. The incremental expression of *UVR8* is accompanied by the up-regulation of key genes in the anthocyanin biosynthesis pathway. In the skin of apples and eggplants, *UVR8* expression increases in response to UV-B, together with a rise in flavonoid and anthocyanin levels. During fruit ripening, UVR8 expression changes (increments and decrements) were reported, suggesting physiological changes do not play a minor role in its regulation (Henry-Kirk et al., 2018; Santin et al., 2019). From this information we can infer that, in the presence of solar UV-B, *UVR8* is involved in the skin coloring of litchi, apple and eggplant throughout fruit development.

Li H. et al. (2018) demonstrated that, in response to UV-B, UVR8 in tomato (SlUVR8) induces the expression of genes and the accumulation of light-absorbing compounds in leaves. Tomato plants containing silenced UVR8 (SIUVR8Ri) presented pale-green fruits when grown under natural sunlight. In contrast, in UVR8 over-expressing plants (SlUVR8OE), the fruits exhibited a darker green color compared to the wild-type (WT). Furthermore, the accumulation of starch, carotenoids, and lycopenes was higher in SlUVR8OE and lower in SIUVR8Ri fruits. The number of chloroplasts per cell was the same in WT and transgenic plants, but plastid size and thylakoid granules increased. The transcriptional factor GOLDEN2-LIKE (GLK2) determines chlorophyll accumulation and chloroplast development in fruits, inducing expression of genes associated to photosynthesis (Waters et al., 2008). When treated with UV-B radiation, SlUVR8 increased both GLK2 accumulation and that of its target genes (Li H. et al., 2018).

Pre and postharvest treatments with UV-B radiation have been employed to induce fruit ripening and pigment accumulation (Castagna et al., 2013). In grape berries, UV-B modifies the quantity and quality of flavonoids, improving their organoleptic properties (Martínez-Lüscherab et al., 2014). UV-B induction of secondary metabolites is not only beneficial to plants but also to human health (Schreiner et al., 2012).

In fruits exposed to UV-B, chloroplasts are larger and starch accumulation is higher, hence influencing the nutritional quality of the fruit in a UVR8 dependent pathway. In addition, it has been observed that, in fruit, UVR8 could induce the synthesis of pigments that absorb UV-B. Since UV-B induces pigment increase and metabolite accumulation, and this impacts directly on production and fruit quality, it would be interesting to examine if these processes are mediated by UVR8.

## Different Inducers of UVR8 Expression

In Arabidopsis, *UVR8* expression increases slightly in response to various abiotic stresses, except for the important increase when osmotic and salt stress are involved (Fasano et al., 2014; Table 3). Expression of the *UVR8* transcript and protein levels increase in Arabidopsis plants grown in MS medium with NaCl and polyethylene glycol (PEG) for 24 h. In *B. platyphylla*, treatments with ABA and NaCl induced UVR8's promotor activity, resulting in an increase in *BpUVR8* transcript levels (Li X. et al., 2018). Expression of *UVR8* gene is induced in foxtail millet cultivar (cv. Prasad) 24 h after salt stress, while in cv. Lepakshi it decreases continuously (Puranik et al., 2011). In leaves of two drought-tolerant barley genotypes (Martin and *Hordeum spontaneum* 41-1 cultivars), *UVR8* transcript levels are higher than in a drought-sensitive genotype (Moroc9-75), but the levels do not change when the three genotypes are exposed to drought stress (Guo et al., 2009). Interestingly, UVR8 expression in barley and millet differ among cultivars of the same species (Guo et al., 2009; Puranik et al., 2011). Under waterlogging conditions, ethylene promotes the formation of lysigenous aerenchyma in the root cortex of *Zea mays*. *ZmUVR8* transcription is induced by waterlogging, mainly in the cortical cells (Rajhi et al., 2011). In radish sprouts, UV-B, cadmium, chilling, and salinity induced the expression of *UVR8* (Wu et al., 2016). Additionally, exogenous addition of H_2_O_2_ and sodium nitroprusside (SNP, an NO donor) also induced *UVR8* expression. This response is inhibited by dimethylthiourea (a H_2_O_2_ scavenger) and cPTIO (2-(4-carboxyphenyl)-4,4,5,5-tetramethylimidazoline-1-oxyl-3 oxide, a NO scavenger) (Wu et al., 2016).

**Table 3:** Regulatory agents of UVR8 expression in different plants.

**Species**	**Gene**	**Regulatory agent**	**Treatment**	**Gene expression** **(X: fold increase of gene expression compared to control)**	**Tissue**	**References**
*Arabidopsis thaliana*	*AtUVR8*	NaCl	MS medium supplemented with 100 mM NaCl for 24 h.	2.5x	Seedling	Fasano et al., 2014
*Arabidopsis thaliana*	*AtUVR8*	Osmotic stress (PEG)	MS medium supplemented with −0.5 MPa PEG for 24 h.	4.5x	Seedling	Fasano et al., 2014
*Arabidopsis thaliana*	*AtUVR8*	Starvation	MS in starvation conditions (no sugar/dark) during 24 h	2x	Seedling	Fasano et al., 2014
*Betula platyphylla*	*BpUVR8*	ABA	10 μM ABA for 24 h	at 3 h (3.3x), 6 h (3.6x), 9 h (4,2x), 12 h (3.25x) and 24 h (1.6x)	3-week-old seedling	Li X. et al., 2018
*Betula platyphylla*	*BpUVR8*	NaCl	100 mM NaCl for 24 h	at 3 h (8.5x), 6 h (14x), 9 h (10.2x), 12 h (6.1x) and 22 h (12.4x)	3-week-old seedling	Li X. et al., 2018
*Camellia sinensis*	*CsUVR8L*	Shading	The nylon black nets with different light transmitting characteristics were placed approximately 1.5 m over the tea plants. The nets were placed when a new round of bud burst started. Tea buds were collected throughout shading treatments.	Significantly decreased at 4 h and 8 h of shading treatment	Tea buds	Liu et al., 2018
*Litchi chinensis*	*LcUVR8* (8 genes)	Light	Samples were collected from 8-year-old litchi. Uncolored fruits were wrapped in pouches and later unwrapped and exposed to light	Expression level increased after the bags were removed	Fruit	Zhang et al., 2016
*Raphanus sativus*	*RsUVR8*	Cadmium (Cd)	Sprouts were subjected to Cd for 12 h in the dark. (Non-specified concentration)	1.3x	Hypocotyl	Wu et al., 2016
*Raphanus sativus*	*RsUVR8*	Chilling	Sprouts were subjected to chilling for 12 h in the dark. (Non-specified temperature)	1.5x	Hypocotyl	Wu et al., 2016
*Raphanus sativus*	*RsUVR8*	NaCl	Sprouts were subjected to NaCl for 12 h in the dark. (Non-specified concentration.)	1.5x	Hypocotyls	Wu et al., 2016
*Raphanus sativus*	*RsUVR8*	H_2_0_2_	Sprouts were subjected to different concentrations H_2_O_2_ (0.2 to 10 mM) for 12 h, and then exposed to white light for another 24 h	up-regulated ranging from 0.5 to 10 mM reaching 2.5x at 5 mM	Hypocotyl	Wu et al., 2016
*Raphanus sativus*	*RsUVR8*	NO	After 36 h dark incubation, the radish sprouts were subjected to 0.5 mM of SNP under white light for 24 h	3.3x	Hypocotyl	Wu et al., 2016
*Setaria italica*	*SiUVR8*	Salinity stress	21-day-old seedlings were treated with 250 mM NaCl for 1, 3, 6, 10, 24 and 48 h. Two cv. tolerant cv. Prasad and sensitive cv. Lepakshi	cv. Prasad: 5x 24 h after stress. cv. Lepakshi: continuously declined over time	Seedling	Puranik et al., 2011
*Solanum lycopersicum*	*SlUVR8*	UV-A	Exposure for 4 h per day to 0.8 J/m2 UV-A (368 nm) for 30 days	2x	Leaf	Mariz-Ponte et al., 2018
*Vitis vinifera*	*VvUVR1*	Temperature	Detached grape berries were exposed to 15 and 35°C	Expression dramatically down-regulated (more than 3 times)	Fruit	Loyola et al., 2016
*Vitis vinifera*	*VvUVR1*	Dark	Detached grape berries were exposed to dark	Expression down-regulated (3 times)	Fruit	Loyola et al., 2016
*Vitis vinifera*	*VvUVR1*	Pathogen infection	Berries infected with *Botrytis cinerea* and leaves infected with *Erysiphe necator* or *Plasmopara viticola*.	Expression down-regulated in berries infected with *Botrytis* and in leaves infected with *Erysiphe*. Expression was up-regulated during latent stages of *E. necator* infection in leaves	Berries and leaves	Loyola et al., 2016
*Vitis vinivera*	*VvUVR8*	White light and UV light	Grape berries just beginning to show color were collected and exposed for 10 days to 15°C/Light (15/L). The light was a mix between white light and UV light with continuous irradiation at 80 μmol·m^−2^s^−1^	Light treatment induced the expression of *UVR8*, whereas dark treatment suppressed this expression	Fruit	Azuma et al., 2015
*Zea mays*	*ZmUVR8L*	Waterlogging and ethylene	12 h under waterlogged conditions with or without pretreatment with an ethylene perception inhibitor 1- ethylcyclopropene (1-MCP), or under aerobic conditions.	Waterlogging induced the expression. The expression induced was blocked by 1-MCP treatment.	Root cortical cells	Rajhi et al., 2011

In *Litchi* fruits, differential expression of multiple *UVR8* genes has been reported. Although the expression of all these genes is induced by light, maximum transcript levels are achieved at different times (Zhang et al., 2016). White light induces the expression of the *UVR8* gene in radish and grape berries, while darkness suppresses it only in grape berries (Azuma et al., 2015; Wu et al., 2016). Loyola et al. (2016), who refer to *VvUVR8* as *UVR1*, showed that UVR8 expression is subject to shading and elevated temperature in *Vitis vinifera* (grape) berries.

As in Arabidopsis, under shading treatment, the expression of *UVR8* does not change significantly in *Camellia sinensis* tea buds. Interestingly, a unigene annotated as *UVR8 LIKE* decreases its expression with shading treatment (Liu et al., 2018).

As previously mentioned, UVR8 gene expression changes in response to UV-B radiation and other agents that provoke several stresses (see Tables 2, 3). Based in this information, we suggest that UVR8 acts as the common factor in the cross-talk among multiple stresses.

## UVR8-independent UV-B Responses

UVR8 is the only UV-B receptor characterized so far (Rizzini et al., 2011), and it has been shown that it mediates a large number of UV-B specific responses (Wargent et al., 2009; Tossi et al., 2014; Li H. et al., 2018). In Arabidopsis leaves exposed to low levels of UV-B, UVR8 regulates the expression of a wide number of key genes in the photomorphogenic and acclimation response. Conversely, under high UV-B doses, the regulation of *WRKY, FAD oxide reductase*, and *UDPgtfp* expression is UVR8-independent (Brown and Jenkins, 2008). The expression of these genes varies in response to several abiotic stresses and is induced by H_2_O_2_ (Inzé et al., 2011; Chen et al., 2012). ARIADNE 12 (ARI12) belongs to a family of proteins with E3 Ubiquitin ligase activity induced by UV-B (Eisenhaber et al., 2007). In low UV-B fluence rate conditions, *ARI12* transcription is induced only in WT and not in the *uvr8* mutants, indicating that gene induction is mediated by the UVR8 receptor. On the contrary, with high UV-B fluence rates, *ARI12* transcript levels increased in both genotypes. Thus, expression of *ARI12* is induced through a UVR8-dependent pathway in low UV-B fluence rate conditions and by an independent one in high fluence rates (Lang-Mladek et al., 2011). Mitogen-activated protein kinase (MAPK) networks are activated by diverse stresses (Holley et al., 2003). Gonzalez Besteiro et al. (2011) demonstrated that UV-B stress activates the signaling cascade mediated by MAPK1 independently from UVR8. When exposed to UV-B doses that cause acute UV-B stress, the MKP1 mutant (*mkp1*) is hypersensitive to radiation, while the *uvr8* mutant is not. On the contrary, at low UV-B doses, the *uvr8* mutant is impaired, but the *mkp1* mutant is not. This indicates that, in response to damaging UV-B doses, MKP1 has a main role, while UVR8 is more important at low levels of UV-B light. Thus, the *mkp1* and *uvr8* mutants allow the genetic dissection of two UV-B response pathways that coordinately determine plant UV-B tolerance.

All this information demonstrates that the participation of UVR8 in the UV-B response is UV-B dose-dependent. UVR8 mediates several responses to low doses of UV-B, while high UV-B doses trigger other adaptive mechanisms. However, other findings suggest there are specific responses to low doses of UV-B that are independent of UVR8. Exposure to UV-B inhibits leaf growth in various plant species (Liu et al., 1995; Searles et al., 2001) due to a reduction in the number of epidermal cells. In both *uvr8-2* and WT plants, the number of epidermal cells per leaf is reduced by UV-B, which suggests that the control of epidermal cell division is independent of UVR8 (Wargent et al., 2009). Moreover, by exposing *uvr8* mutants to outdoor conditions, it was observed that UV-B radiation affected morphology by a UVR8-independent mechanism (Coffey et al., 2017). At the DNA level, UV-B radiation induces cyclobutane pyrimidine dimer (CPD) formation, which inhibits transcription and replication, and induces mutations. The activity of CPD photolyases is thus essential for protecting genome integrity from UV-B radiation. Expression of the CPD photolyase (*PHR*) gene is mediated by both UVR8-dependent and UVR8-independent pathways. In *uvr8-6* mutants, the expression of *AtPHR* is induced by low doses of UV-B, which generate CPDS and not ROS (Li et al., 2015).

These responses, which appear at UV-B doses that do not produce oxidative stress and are independent of UVR8, would suggest the existence of new and/or different UV-B receptor/s.

## Discussion

### UVR8 in Green Algae, Bryophytes and Angiosperms

The UV-B light receptor has been thoroughly characterized in Arabidopsis since Kliebenstein first identified it in 2002 (Kliebenstein et al., 2002). Over the last 4 years, research on UVR8 has been conducted for other plant species such as apple, tomato, grape, etc. (Loyola et al., 2016; Zhao et al., 2016; Li H. et al., 2018). Employing bioinformatic analyses, the protein's functional motifs have shown to be widely conserved in green algae, bryophytes, lycophytes and angiosperms (Fernández et al., 2016), but it has not been found in gymnosperms yet. Fernández et al. (2016) suggest that this might be due to the absence of whole genome sequences for gymnosperm species. Gymnosperms are more tolerant than Angiosperms to UV-B injury because their epidermal cells and leaf anatomy are more effective at attenuating UV-B radiation (Bassman et al., 2001). In some outdoor-grown gymnosperms, such as Scots pine and Norway spruce, UV-B radiation has no significant effects on growth or secondary compounds (Turtola et al., 2006). Based on this information, we propose that UVR8 sequences in gymnosperm have not been detected because either the receptor is unnecessary for this group or there is another receptor.

The UVR8 gene copy number changes between plant species. Chlorophytes contain a single copy of *UVR8*, same as in Arabidopsis, but bryophytes contain two copies. As in bryophytes, 40% of monocot species contain two UVR8 copies and 32% of dicots contain more than one *UVR8* copies (Brown et al., 2005; Fernández et al., 2016). The difference in the *UVR8* gene copy number among plant species is an interesting subject of debate. There are several articles that show that an increase in gene copy number and polyploidy contribute in the adaptation to stress (te Beest et al., 2011; Soltis et al., 2015; Panchy et al., 2016).

Chlorophytes mark the first appearance of the *UVR8* gene in the *Viridiplantae* kingdom (Fernández et al., 2016). Chlorophytes live in water bodies where UV-B is 100% filtered within the first meter of depth (Zellmer, 1995). This group is able to float, thus allowing green algae to regulate the UV-B doses to which they are exposed. The bryophytes were the first terrestrial plants; they were exposed to high UV-B doses and could not avoid the radiation. In this condition, the presence of two *UVR8* gene copies could give them an additional UV-B stress tolerance. When higher plants colonized the earth, some of them grew in places characterized by high UV-B doses and others by low UV-B doses. Under this heterogeneous scenario, the loss or gain of a gene copy could be an adaptive advantage. Interestingly, a study of the genome of the marine Angiosperm *Zostera marina* claims that *UVR8* is not present (Olsen et al., 2016). This plant is found in mostly shallow coastal soft bottom environments with reasonably high water clarity to allow growth at a depth enough to filter almost all UV-B light (Short and Coles, 2001).

It will be interesting to evaluate a potential correlation between the number of *UVR8* gene copies and the tolerance to UV-B stress or even the diversity of different eco-geographical niches a species can colonize. Elucidating if higher copy numbers of *UVR8* are linked to an increase in the protein levels and if the multiple copies have redundant roles are also important challenges.

At the protein level, UVR8 has been characterized in Arabidopsis and in others plants species with similar results. However, contrary to Arabidopsis, in *M. polymorpha*, the UVR8 receptor is mainly present as a monomer in absence of UV-B, probably due to the poor structural stability of the dimer (Soriano et al., 2018). Furthermore, the subcellular localization differs from Arabidopsis, with MpUVR8 found constitutively in the nucleus (Soriano et al., 2018). Despite these differences, both proteins are functional.

Functional motifs that regulate protein stability and localization are conserved between species (Soriano et al., 2018). The observed differences could be attributed to small variations in amino acid sequences localized outside these motifs. This information suggests that amino acid sequences important to UVR8 behavior could remain unidentified.

### UVR8 Gene Is Differentially Expressed in Plant Organs and Throughout Fruit Development

*UVR8* expression is ubiquitous in every species analyzed so far (Rizzini et al., 2011; Jenkins, 2017; Table 1). Although in some species *UVR8* expression is constitutive, in other species the expression varies in a tissue- and development-dependent fashion (Table 1). In fruits, for example, UVR8 expression is induced during development (Table 1). Some plant species have several UVR8 genes, e.g., litchi fruits (Fernández et al., 2016; Zhang et al., 2016). The UVR8 gene copy number could account for the differential expression observed in this plant. It will be thus interesting to analyze differences in the relative levels of either UVR8 transcripts during development.

*UVR8* is expressed and UVR8 protein is detected in all plant organs (Rizzini et al., 2011). Transcript levels are higher in leaf, flower and fruit than in root (Table 1). This difference between plant organs could be associated with the necessity of the plant to protect itself from UV-B. On the other hand, the detection of *UVR8* transcript in root, an organ that does not receive UV-B light, suggests other functions for UVR8 beyond light perception. Recently, using a *Nicotiana* silenced roots (irNaUVR8) approach, it has been demonstrated that UVR8 mediates colonization of *Deinococcus* (Santhanam et al., 2017).

Throughout fruit developmental stages, UVR8 transcript levels change (Table 1), indicating the possible participation of UVR8 in this process. Li H. et al. (2018) have determined that UVR8 improves chloroplast development in the fruit through the regulation of SlGLK2 in tomato grown outdoors. The study of UVR8 in different species has allowed the identification of new UVR8 functions demonstrating that UVR8 not only participates in the perception of UV-B radiation.

### UV-B Regulates UVR8 Transcript Expression in Different Plant Species

*UVR8* expression is constitutive in Arabidopsis plants exposed to UV-B (Kaiserli and Jenkins, 2007; Fasano et al., 2014). However, changes in the levels of the *UVR8* transcript were detected in several plants treated with UV-B (Table 2). The levels of UVR8 transcripts induced by UV-B increase in the first hours of treatment, but later decrease (Table 2). In *Zea mays*, UVR8 expression was transient and also systemic in shielded leaves of UV-B exposed plants (Casati et al., 2011a). This information suggests the existence of a mechanism regulating *UVR8* gene expression by UV-B.

*UVR8* transcript level induction is an energetically efficient mechanism in most plants, since the receptor is synthesized only when required. This, however, implies a slower adaptation to UV-B acute irradiation. In *Arabidopsis*, in turn, constitutive expression and dynamic regulation, which depends on the UVR8 dimer-monomer equilibrium, allows for an immediate –although more energetically expensive- response (Rizzini et al., 2011). Selection of one strategy in lieu of the other could be due to a difference in tolerance to UV-B and, in some instances, both modes of UV-B acclimation could be active together, depending on the levels of perceived radiation.

RUP1 and RUP2 are negative feedback regulators of the UV-B signaling cascade. Both proteins interact with UVR8-COP1, facilitating UVR8 redimerization (Gruber et al., 2010; Heijde et al., 2013). In monocotyledons, we found RUP2 sequences with percentages of similarity lower than 50% compared to Arabidopsis, but no sequences similar to RUP1 (Figure 2). In this group, UVR8 activity regulation could be a balance between UVR8 synthesis and degradation and re-dimerization mediated by RUPs proteins would not be necessary. Nonetheless, the coexistence of both mechanisms of UVR8 activity regulation in some plants cannot be ruled out.

### UVR8 Participates in Response to Multiple Stresses

Several reports have demonstrated that UVR8 participates in diverse stresses, often in combination with UV-B (Demkura and Ballaré, 2012; Fasano et al., 2014; Santhanam et al., 2017). The metabolites produced in UV-B response mediated by UVR8 are beneficial when the plant faces other stresses (Demkura and Ballaré, 2012). As shown in Tables 2, 3, UVR8 expression is induced by UV-B, salinity, starvation, pathogen infection, waterlogging, UV-A, chilling, etc. The regulation of *UVR8* transcript levels in response to different stresses suggests a possible participation of the receptor. The UVR8 transcript expression in leaves of two barley genotypes tolerant to drought are higher in cultivars *Martin* and *Hordeum spontaneum* 41-1 compared to a genotype sensitive to drought (Moroc9-75) (Guo et al., 2009). The correlation between the UVR8 expression levels—stress tolerance, and the evaluation of the role of UVR8 in those stresses have not yet been determined.

ABA plays an important role both in plant development and in response to abiotic stresses (Tuteja, 2007; Trivedi et al., 2016). In plants exposed to high doses of UV-B, ABA production is induced, activating NADPH oxidase and generating H_2_O_2_, and also increasing NO production, which abates the damage caused by UV-B (Tossi et al., 2009, 2012). In radish hypocotyls, UV-B, H_2_O_2_ and nitric oxide (NO) induce UVR8 expression, whereas a chemical trap for H_2_O_2_ represses it (Wu et al., 2016). UVR8 transcript expression is induced by salinity, chilling and osmotic stress (Table 3). Interestingly, these stresses are regulated by ABA (Tuteja, 2007). Treatments with ABA, salt stress, high temperatures and UV-B increase the activity of the BpUVR8 promoter in transgenic tobacco leaves (Li X. et al., 2018). ABA could play a key role in the induction of *UVR8* expression in abiotic stresses and development. The interaction between ABA and UVR8 should be thoroughly examined. The identification of specific motives in UVR8 promoters of different species could be an approach to identify other regulatory agents of UVR8 transcript expression.

## Conclusion

The advantage of working with Arabidopsis is evident and, in this particular case, it has facilitated the characterization of UVR8. However, results gathered from different plant species suggest that the results obtained with Arabidopsis cannot be extended wholly to all plants due to the extant diversity. The study of UVR8 physiology in several species beyond Arabidopsis has enabled to establish that:

- UVR8 is highly conserved and is functional in green algae, bryophytes and angiosperms.
- There are species that present more than one copy of *UVR8* gene, and in some species the gene can undergo alternative splicing, generating two UVR8 functional proteins.
- The UVR8 gene is expressed differentially in different organs and throughout fruit development.
- UVR8 is involved in several pathways other than UV-B perception. In tomato fruit, for instance, UVR8 regulates the development of the chloroplast and the synthesis of chlorophyll, carotenoids, starch and lycopenes, thus influencing its nutritional quality. In Nicotiana roots, it mediates colonization by Deinococcus.
- UVR8 transcript expression is regulated by UV-B, H_2_O_2_, ABA and other stresses.

The information obtained using different plant species show that UVR8 is a versatile molecule involved in perception of UV-B, as well as in developmental and stress processes. However, there is a lack of knowledge regarding UVR8 activity and its importance in these responses. Some important questions that arise are, among others, the following:

- How is UVR8 regulated during these processes?
- Is there a correlation between the UVR8 transcript levels and stress tolerance?
- How does negative feedback work in plant species where RUP 1 has not been detected and RUP2 has a low similarity percentage?
- How does UVR8 participate in developmental processes under natural growth environments?

Only a comprehensive consideration of UVR8 physiology in different species will allow us to bring light into the mechanisms and importance of this key modulator of several aspects involving plant evolution, development and survival. In addition the information derived from the analysis of the conservation and/or the variability of the responses in different plants might be harnessed to improve cultivar management by means of biotechnological approaches.

## Author Contributions

VT and JR conceived the work and wrote the manuscript, except sections UVR8: from green algae to higher plants and UVR8 in fruit developmental stages. JI wrote section UVR8 in fruit developmental stages UVR8-independent UV-B responses. LL contributed in writing the Discussion and reviewing critically the manuscript. HB wrote section UVR8: from green algae to higher plants UVR8: from green algae to higher plants. AE supervised the writing of the manuscript. SP-Á coordinated the work and carried out the native English edition of the manuscript.

### Conflict of Interest Statement

The authors declare that the research was conducted in the absence of any commercial or financial relationships that could be construed as a potential conflict of interest.
